# Features of Cancer mHealth Apps and Evidence for Patient Preferences: Scoping Literature Review

**DOI:** 10.2196/37330

**Published:** 2023-04-28

**Authors:** Shannon Vaffis, Soluna Whaley, David Rhys Axon, Elizabeth Hall-Lipsy, Ana Hincapie, Marion Slack, Terri Warholak

**Affiliations:** 1 College of Pharmacy University of Arizona Tucson, AZ United States; 2 James L Winkle College of Pharmacy University of Cincinnati Cincinnati, OH United States

**Keywords:** scoping review, mHealth, mobile health, health app, cancer, oncology, disease self-management, self-management, chronic disease, tablet, smartphone, digital health, eHealth, feature

## Abstract

**Background:**

Cancer is increasingly being treated as a chronic disease rather than an acute one-time illness. Additionally, oral anticancer therapies, as opposed to intravenous chemotherapy, are now available for an increasing number of cancer indications. Mobile health (mHealth) apps for use on mobile devices (eg, smartphones or tablets) are designed to help patients with medication adherence, symptom tracking, and disease management. Several previous literature reviews have been conducted regarding mHealth apps for cancer. However, these studies did not address patient preferences for the features of cancer mHealth apps.

**Objective:**

The primary aim was to review the scientific literature that describes the features and functions of mHealth apps designed for cancer self-management.

**Methods:**

As the purpose of this review was to explore the depth and breadth of research on mHealth app features for cancer self-management, a scoping review methodology was adopted. Four databases were used for this review: PubMed/MEDLINE, Embase, CINAHL, and PsycINFO. Citation and reference searches were conducted for manuscripts meeting the inclusion criteria. A gray literature search was also conducted. Data extracted from manuscripts included author, title, publication date, study type, sampling type, cancer type, treatment, age of participants, features, availability (free or subscription), design input, and patient preferences. Finally, the features listed for each app were compared, highlighting similarities across platforms as well as features unique to each app.

**Results:**

After the removal of duplicates, 522 manuscripts remained for the title and abstract review, with 51 undergoing full-text review. A total of 7 manuscripts (referred to as studies hereafter) were included in the final scoping review. App features described in each study varied from 2 to 11, with a median of 4 features per app. The most reported feature was a symptom or side effect tracker, which was reported in 6 studies. Two apps specified the inclusion of patients and health care providers during the design, while 1 app noted that IT and communications experts provided design input. The utility of the apps for end users was measured in several ways, including acceptability (measuring the end users’ experience), usability (assessing the functionality and performance by observing real users completing tasks), or qualitative data (reports from end users collected from interviews or focus groups).

**Conclusions:**

This review explored the literature on cancer mHealth apps. Popular features within these mHealth apps include symptom trackers, cancer education, and medication trackers. However, these apps and features are often developed with little input from patients. Additionally, there is little information regarding patient preferences for the features of existing apps. While the number of cancer-related apps available for download continues to increase, further exploration of patient preferences for app features could result in apps that better meet patient disease self-management needs.

## Introduction

Cancer is increasingly being treated as a chronic disease rather than an acute one-time illness [1-3]. Some cancers, such as chronic leukemia and ovarian cancer can be managed, sometimes described as “controlled,” in a state where the cancer does not grow but is also not cured for months or years. Additionally, oral anticancer therapies, as opposed to intravenous chemotherapy, are now available for an increasing number of cancer indications [4,5]. These oral treatments are typically self-administered by the patient outside of the clinical setting, presenting challenges (eg, symptom and side effect management) for patients, their families, and their caregivers [6-8].

A 2015 literature review found that health care systems and patients were meeting the challenges of managing self-administered medicines by using mobile health (mHealth) software apps [9]. mHealth apps for use on mobile devices (eg, smartphones or tablets) are designed to help patients with medication adherence, symptom tracking, and disease management [10]. A 2021 analysis found 794 oncology-specific English language mHealth apps [11]. Nasi et al [9] found that patients with cancer mainly used mHealth apps for self-management activities. Self-management can be described as a patient’s ability to deal with all aspects of a chronic illness, such as symptoms; treatments; and physical, social, and lifestyle changes.

A wide variety of mHealth apps are available for cancer care (prevention, screening, diagnosis, treatment management, and survivorship) [12,13]. While some apps allow for two-way communication with health care professionals or caregivers, others are solely for the patient to track data such as disease symptoms or physical activity [12,14]. A literature review conducted by Bender et al [15] cataloged mHealth apps providing tools for the self-management of cancer and sorted their features into three groups: appointment tools (eg, reminders for visits with the health care team), self-monitoring functionality (eg, patient tracking of disease symptoms and medication side effects), and communication capability (eg, SMS text messaging with a member of the health care team). With such heterogeneity in functionality, it is imperative to understand what features are preferred by patients to best meet their cancer care needs.

Smartphone ownership in the United States has reached at least 81% according to the Pew Research Center [16], bringing mHealth apps to a majority of the adult population. However, in 2012, a study by Pandey et al [14] showed that fewer than half of cancer care apps were free of cost (42.8%), while the remainder charged fees for downloading. As such, access to mHealth apps remains an important consideration when assessing whether they can aid patients in disease self-management.

Several previous literature reviews have been conducted regarding mHealth apps for cancer. Bender et al [15] conducted a systematic review and content analysis of apps for the prevention, detection, and management of cancer. Nasi et al [9] conducted a literature review regarding the role and use of mHealth technologies during the cancer care process with a particular focus on supportive care. Davis and Oakley-Girvan [13] conducted a literature review to identify apps across the cancer care continuum (from prevention to survivorship) examining patient education and recommendations from randomized studies. Pandey et al [14] evaluated the availability and content of apps for patients with cancer. Finally, Tabi et al [17] reviewed medication management apps for oncology patients. However, these studies did not address patient preferences for the features of cancer mHealth apps.

Our primary objective was to review the scientific literature that describes the features and functions of mHealth apps designed for cancer self-management.

## Methods

### Overview

This review used a scoping literature review methodology. As stated by Munn et al [18], a systematic review is indicated when the purpose of the research is to compare clinical practices or inform decision-making, whereas a scoping review is indicated when the purpose of the review is to explore how research in the field is conducted and the kinds of literature available. As the purpose of this review was to explore the depth and breadth of research on mHealth app features for cancer self-management, a scoping review methodology was adopted. Guidance was drawn from several sources including the seminal Arksey and O’Malley [19] article, the Tricco et al [20] scoping review guidelines, the McGowan et al [21] PRISMA-ScR (Preferred Reporting Items for Systematic Reviews and Meta-Analyses Extension for Scoping Reviews), and the Peters et al [22] updates to the Joanna Briggs Institute Guidelines. The reporting in this manuscript follows the PRISMA-ScR extension guidance. This review protocol was not registered. The corresponding author may be contacted regarding the protocol.

### Inclusion Criteria

This review included manuscripts related to patient preference studies for cancer self-management using mHealth apps; utilization studies for cancer self-management mHealth apps; utility analyses for cancer self-management mHealth apps; and gray literature from web-based or trade publications related to consumer preference for, use of, or utility for cancer self-management mHealth software apps. Only studies for adults diagnosed with cancer were included. No limits were placed on the type of study considered for inclusion (eg, experimental vs descriptive).

### Exclusion Criteria

Manuscripts not written in English were excluded. Pediatric studies were not included. Studies that focused on app development for cancer prevention, diagnosis, palliative care, or survivorship support were not included. Additionally, manuscripts published before 2010 were not included as technology evolutions would likely have rendered previous apps obsolete [23].

### Search Strategy

Four databases were used for this review: PubMed/MEDLINE, Embase, CINAHL, and PsycINFO. The database searches were conducted between February 1 and April 1, 2021. A protocol was developed a priori outlining search strategies including databases, websites, and search terms. Exploratory searches were conducted in PubMed and Google Scholar to gather potential search terms. Manuscripts from the exploratory searches were reviewed, and keywords were collated to begin building a search strategy. Once a successful search strategy was built in PubMed, the Polyglot Search Translator was used to build additional searches for the other three databases [24]. The final search strategy for PubMed is presented in Textbox 1. Citation and reference searches were conducted for manuscripts meeting the inclusion criteria. A gray literature search was also conducted across technology trade publications (eg, HealthTech Magazine) and health professional organization publications (eg, American Society for Clinical Oncology and International Society for Pharmaceutical and Outcomes Research).

**PubMed search strategy.**

(“Neoplasms”[Mesh] OR “cancer”[ALL] OR “oncology”[ALL] OR “neoplasm*”[ALL]) AND (“Patient Preference”[Mesh] OR “Patient Satisfaction”[Mesh] OR “acceptability”[ALL] OR “utility”[ALL] OR “patient preference”[ALL] OR “patient satisfaction”[ALL] OR “usability”[ALL]) AND (“Telemedicine”[Mesh] OR “User-computer Interface”[Mesh] OR “mobile health”[ALL] OR “mHealth”[ALL] OR “mobile application”[ALL] OR “smart phone application”[ALL] OR “mobile app”[ALL] OR “smart phone app”[ALL] OR “smartphone application”[ALL] OR “smartphone app”[ALL]) AND (“Self-Management”[Mesh] OR “Self Care”[Mesh] OR “Treatment Adherence and Compliance”[Mesh] OR “Patient Compliance”[Mesh] OR “self-management”[ALL] OR “adherence”[ALL] OR “disease self-management”[ALL] OR “cancer supportive care”[ALL])

### Data Extraction

The research team developed title/abstract screening and full-text review forms based on the inclusion and exclusion criteria above. Two independent reviewers (SV and SW) completed the title/abstract screening and full-text review forms for the peer-reviewed and gray literature. If consensus was not reached between the two reviewers, a third independent reviewer (a senior member of the research team) provided arbitration.

Data were extracted from the manuscripts meeting the inclusion criteria and collated in Excel (2017; Microsoft Corporation). Data extracted from manuscripts included author, title, publication date, study design, sampling type, cancer type, treatment, age of participants, features, availability/cost (free or subscription), design input, and patient preferences. One or more members of the research team verified the accuracy of the tabularized data and resolved any discrepancies. Finally, the features listed for each app were compared, highlighting similarities across platforms as well as features unique to each app.

### Ethical Considerations

This review was deemed to be not human subjects research by the University of Arizona Internal Review Board.

## Results

### Overview

**Figure 1:**
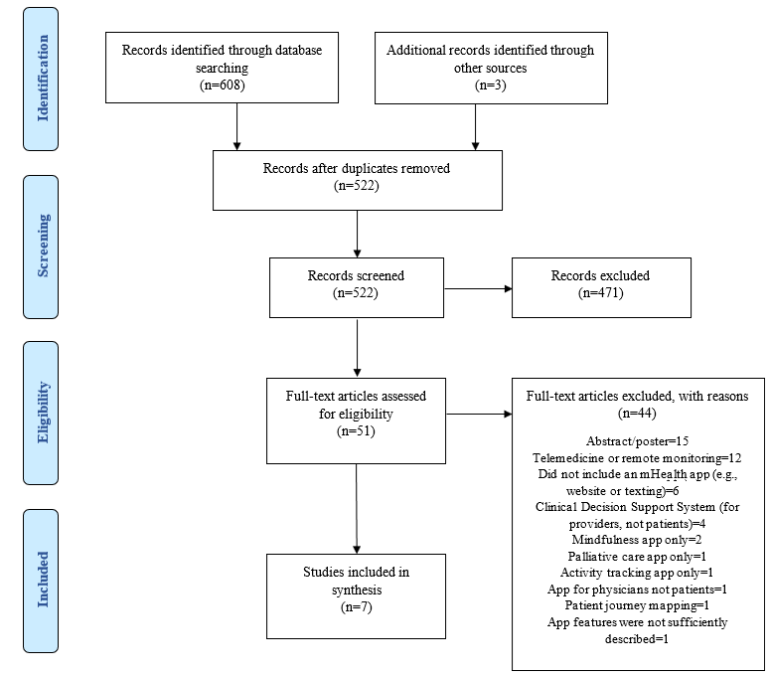
PRISMA (Prefered Reporting Items for Systematic Reviews and Meta-Analyses) flow diagram of the record selection process. mHealth: mobile health.

The initial search identified 611 manuscripts. After the removal of duplicates, 522 manuscripts remained for the title and abstract review, with 51 undergoing full-text review. A total of 7 manuscripts (referred to as studies hereafter) were included in the final scoping review. The outcomes of the database searches, title and abstract reviews, and full-text reviews (as well as reasons for exclusion) are presented in a PRISMA flow diagram of the manuscript selection process (Figure 1). Data extracted from the scoping review are presented in Table 1 and Multimedia Appendix 1 [25-31].

**Table 1:** Features of mHealth apps in the scoping literature review.

Features	Birkhoff et al [25]	Fishbein et al [26]	Greer et al [30]	Jacobs et al [27]	Kongshaug et al [28]	Tran et al [29]	Wang et al [31]
Symptom tracker	✓^a^	✓	✓		✓	✓	✓
Emotional/social well-being	✓			✓			
Medication tracker	✓		✓				
Reminders		✓	✓		✓	✓	✓
Tools and settings	✓			✓			
Landing page		✓		✓			
Education		✓	✓				✓
Health and fitness		✓	✓	✓			
Calendar	✓				✓		
Medical/treatment information				✓			✓
Privacy/data use		✓					
Notes and questions		✓					
Personalized dose schedule			✓				
Journaling	✓						
To-do list	✓			✓			
Weight tracking	✓						
Patient decision support					✓		
Vital sign tracking	✓						

^a^Indicates presence of the feature.

### Study Design and Publication Date

While 5 of the included studies were descriptive [25-29], 1 study was experimental [30] and 1 study was quasi-experimental [31]. The descriptive studies used a variety of methodologies. Three were feasibility studies including combinations of app trials, patient interviews, and expert focus groups [25,28,29]. Two of the descriptive studies were usability tests including measures of acceptability or barriers [26,27]. The experimental study compared the improvement of symptoms and medication adherence between two patient groups (using app vs standard care) [30]. The quasi-experimental study compared patient care needs (eg, psychological support and communications with the care team) between two patient groups, one of which received routine care and one with access to the patient app [31]. Publication dates ranged from 2017 to 2021.

### Sample Size

Sample sizes of included studies varied widely, ranging from 11 to 181, with descriptive studies including smaller samples and the quasi-experimental and experimental studies including 100 and 181 patients, respectively. Most studies, including the experimental and quasi-experimental studies, used convenience sampling [25,26,29-31] or did not cite sampling methodology [27,28].

### Cancer Type

Four apps were developed to support a single subpopulation of patients with cancer such as breast [27], gastrointestinal [28], oral [31], or prostate cancer [29]. The remaining apps were designed to serve a diverse cancer patient population, including 1 app that was designed to support a wide range of diseases such as asthma and cardiac health [25,26,30]. Three apps were designed to support oral chemotherapy treatment regimens [26,28,30]. Two apps were designed to support mixed treatment regimens [27,29]. One app each was designed to support radiation [25] or surgical treatment [31].

### Age of Participants

Four studies reported a mean age for participants (mean age ranged from 52 to 57 years) [25,27,30,31], and 1 study reported a median age of 55 years [27]. One study reported only an age range from 40 to 79 years [28], and 1 study did not specify participant ages [26].

### App Features

App features described in each study varied from 2 to 11, with a median of 4 features per app. The most reported feature was a symptom or side effect tracker, which was reported in 6 studies [25,26,28-31]. While there were 5 emotional/social support features reported, they were found in only 2 apps. “Circle of support” and “Healthy dose” functionality were reported by Birkhoff et al [25], and “Social support,” “Emotional support,” and “Local resources” (which provided users with contact information for emotional and social support services in their community) were reported by Jacobs et al [27]. A total of 20 different types of app features were reported ranging from a home page and settings to medication adherence trackers and calendars. A total of 5 features were unique to single apps: notes and questions [26], notices of privacy and data use [26], personalized medication dosing schedule (with optional reminders) [30], vital sign tracker [25], and weight tracking [25].

### Availability/Cost

Two apps were noted to be free and publicly available for download [25,27], 2 were only available to study participants or the patients of a particular cancer treatment facility at the time of publication [30,31], and the remainder did not specify availability [26,28,29].

### Design Input

Three apps specified the inclusion of patients and health care providers during the design [26,27,30], while 1 app noted that the IT and communications experts provided design input [28]. The remainder did not specify [25,29,31].

### Measure of Acceptability

The utility of technology for end users can be measured in several ways, including acceptability (measuring the end users’ experience), usability (assessing the functionality and performance by observing real users completing tasks), or qualitative data (reports from end users collected from interviews or focus groups). In the study by Birkhoff et al [25], both usability and acceptability were reported. The overall usability score was 4.69 out of 7, though considerably higher among high school–educated patients (6.38) versus graduate degree–educated patients (3.87). There was no significant difference in reported use over time. In the study by Jacobs et al [27], acceptability was reported as a usefulness score (4.2/5); while engagement with the app over the study period was high, several improvements were suggested qualitatively, such as greater integration with local support services. The study by Wang et al [31] reported acceptability among the intervention group over time. Baseline (odds ratio) scores were reported for intention to use (2.54), perceived usefulness (2.52), and perceived ease of use (2.32) compared to postintervention scores of 3.02, 2.95, and 3.01, respectively, a significant increase in all three aspects. Three studies presented utility as qualitative data [26,28,29]. Fishbein et al [26] noted that usability and acceptability tests were performed but not reported, reporting instead that stakeholder feedback had been incorporated into the design from focus groups and alpha and beta testing, as this was an app design protocol. Kongshaug et al [28] reported that the app provided patients with reassurance regarding correct oral chemo treatment, the app was used as a memory tool for discussing medication adherence and side effects with the health care team, and patients were concerned about reporting less serious side effects. In addition, health personnel expressed a positive attitude to integrate the tool into everyday work. Tran et al [29] reported that patients valued the emotional and well-being support over symptom reporting, requested incorporating patient web-based communities of support (eg, Facebook or Reddit), were concerned with future data use and privacy, and requested data summary features to help them track the information they were entering over time. Finally, Greer et al [30] did not report usability, acceptability, or qualitative data.

## Discussion

### Principal Findings

In total, 7 studies published from 2017 to 2021 were included for analysis. Studies varied in methodology, from descriptive to experimental, and size, with subject sizes ranging from 11 to 181. Additionally, apps were developed to address the needs of a heterogeneous patient population, some address the needs of a single cancer indication or treatment, and others provide support across the spectrum of cancer diagnoses. Likewise, the number of features per app varied from 2 to 11 with a median of 4—with the most reported feature being a symptom tracker. Lastly, several studies reported patient acceptability or preference data for the app or the features, with acceptability (assessed through survey or interviews) most frequently reported.

Our objective was to review the features and functions of mHealth cancer self-management apps. Symptom tracking, education/information, and medication tracking were three of the most frequently reported features, each of which is discussed in turn below.

A symptom tracker was the most reported feature across the manuscripts in this review, reported in 6 of 7 manuscripts. Cooley et al [32] noted that symptom tracking (particularly with eHealth applications) was relevant to improved patient outcomes in cancer treatment. Similar results were shown by Lu et al [33] who conducted a systematic review to evaluate the use of mHealth apps to track patient-reported cancer outcomes such as symptom reporting. Their search of the iOS Apple Store and Android Google Play identified 11 cancer-specific apps with symptom tracking features. Further details of these features were explored. Some symptom trackers offered the ability for patients to add symptoms not already listed, record symptom severity, add notes, provide a graphical summary, or export data to a caregiver or health professional. Two apps in our review were able to provide symptom trend reports and graphical information [26,28], but only 1 specifically noted the ability to log symptom severity [26]. Further studies may seek to examine patient preferences for symptom trackers, such as the utility derived from displaying symptom reporting trends over time.

This review found that patient education features were reported in 3 studies [26,30,31]. Similarly, Richards et al [34] explored the importance of patient education within mHealth apps, conducting a systematic review to assess how patients used their mobile devices to access information to support outpatient disease management. A total of 14 different interventions were identified across 23 published studies. The education-related features described by Richards et al [34] were related to treatment and did not meet the full range of patient information needs regarding treatments and symptom management. In contrast, the education features identified in our review attempted to meet a broader spectrum of information needs including symptom management and other cancer-related topics (eg, nutrition). Likewise, 3 of the studies included in this review included a home page (at least one of which provided health recipes and news items). Finally, our review identified a total of 5 emotional or social support features that were reported within 2 apps (including information on local patient and caregiver support groups and services) [25,27]. While many of the app features were not described as primarily providing cancer care information, several of the features included information to support patients with disease self-management.

Medication trackers were not typical offerings for cancer self-care apps included in our review, as they were present in only 2 studies. Similarly, Skrabal Ross et al [35] conducted a scoping review to better understand mobile phone apps that were designed to enhance medication adherence to oral chemotherapy. Skrabal Ross et al [35] identified 5 studies with electronic medication adherence interventions; however, only 2 used an mHealth app (the others were SMS text message based). Alarms and reminders were used in both apps to increase patient medication adherence. Likewise, alerts and reminders were identified in 4 apps in our review [28-31]. Like our review, none of the apps included in the study by Skrabal Ross et al [35] were noted to contain a feature for tracking medication-taking behavior trends over time. A study by McNamara et al [36] noted the difficulty in managing patient oral oncolytic medication adherence, and an article by Burhenn and Smudde [37] advocates for tools (eg, smartphone apps) to aid patients in medication adherence. Therefore, further research is warranted to explore whether medication tracking features of mHealth apps aid in medication adherence for patients with cancer treated with oral oncolytic medication.

Despite the growing number of oncology apps, challenges of access do remain for patients seeking to use mHealth for cancer self-management. Our review noted that several apps were available only to patients of a particular cancer center or health system. Similarly, a study by Ana et al [38] noted that, while there are an increasing number of clinical trials aimed at increasing patient medication management through the use of an mHealth app, many of these apps are removed from app stores after the trial ends. Thus potential resources remain out of patient reach.

### Limitations

This was a scoping review rather than a systematic review; therefore, a quality assessment was not conducted for the studies meeting the inclusion criteria. Future research could consider conducting a systematic review; assessing the quality of the studies included in the review may lead to further insights. This review specifically sought information on smartphone apps—not SMS text messaging or web-based apps. Accordingly, a narrow range of inclusion dates was used to account for current smartphone operating systems. While not a specific inclusion criterion, patient preference was an area of research interest, and not all studies included reported such.

### Future Research

The information found in our review may be of value as cancer apps are continuously developed and updated. Researchers have not always used the preferences of patients in the design of apps. Many of the app features identified in this review included optional calendar reminders, alerts, or trend graphs, although how useful patients find these optional functions is less clear. Additionally, there may be key features that would enhance use that are yet undiscovered.

Further assessment of available features should be conducted among subject matter experts in the fields of mHealth cancer app development and cancer clinical care to explore whether the features currently available are useful and relevant for patients (ie, meet patient preferences). This may enable the development of mHealth apps that better meet patient needs for disease self-management, both from a technical and clinical perspective. Further clarity is needed regarding whether currently available features are used by patients. In addition, some features are heterogeneous across apps. For example, some medication trackers also feature optional alerts when medication should be taken or reminders to track medication adherence, but it remains unclear how many patients use these options or how often. This information could be transformed into a discrete choice experiment to better understand patient preferences for app features. Lastly, this can inform future app development or existing app revision.

### Conclusions

While the number of cancer-related apps available for download continues to increase, further exploration of patient preferences for app features could result in apps that better meet patient disease self-management needs. Currently, there is a lack of consensus regarding the presentation of information on patient input into the app design process; reporting best practices may increase the comparability of research. Patient access to cancer self-management apps remains limited. Future research may also include the evaluation of mHealth apps upon development completion from an end user (patient) perspective.

## Appendix

Multimedia Appendix 1 Study characteristics.

Multimedia Appendix 2 PRISMA (Preferred Reporting Items for Systematic Reviews and Meta-Analysis) checklist.

## Abbreviations

mHealth: mobile health
PRISMA-ScR: Preferred Reporting Items for Systematic Reviews and Meta-Analyses Extension for Scoping Reviews
