# Supplementary material for: Features of Cancer mHealth Apps and Evidence for Patient Preferences: Scoping Literature Review
Source: JMIR Cancer. 2023 Apr 28;9:e37330. doi: 10.2196/37330 (PMC10182455; doi:10.2196/37330)
Supplement: Multimedia Appendix 1 [file cancer_v9i1e37330_app1.docx]

| **First Author** | **Publication**  **Date** | **Study type** | **Sample size** | **Sampling type** | **Cancer**  **type** | **Treatment** | **Age of participants** |
| --- | --- | --- | --- | --- | --- | --- | --- |
| **Birkhoff** | 2018 | Descriptive - mixed methods feasibility (quantitative and qualitative) | 60 recruited 32 completed | Convenience - sample of radiation oncology patients | not specified | Radiation | 53.59 (Mean) 13.687 (SD) 22-77 (Range) |
| **Fishbein** | 2017 | Descriptive - evaluation of usability and acceptability | 32 stakeholders  Patient/families Oncology clinicians Cancer practice administrators Representatives of health system, community, and society) | Stakeholders were included as consultants (not participants) so no descriptive statistics are shown | Diverse cancer population | Oral chemotherapy | Not specified |
| **Greer** | 2020 | Experimental - randomized trial | 91-mobile app 90-standard care | Convenience - patients receiving treatment at Massachusetts General Hospital Cancer Center (or 2 satellite sites) | Diverse cancer population | Oral chemotherapy | 53.30 (Mean) 12.91 (SD) 21-88 (Range) |
| **Jacobs** | 2019 | Descriptive - usability and barriers | 4 breast cancer survivors 7 healthcare professionals (cancer navigators and oncology nurses) | Not specified | Breast | Multiple: Surgery  Radiation Chemotherapy Hormone therapy | Average 52 |
| **Kongshaug** | 2021 | Descriptive - feasibility (app trial, patient interviews, healthcare professional focus groups) | 14 patients recruited 9 patients completed (and were interviewed) 7 oncologists 7 oncology nurses | Not specified | Gastrointestinal cancer | Oral chemotherapy | ~40-79 (Range) |
| **Tran** | 2020 | Descriptive - feasibility (app trial, patient interviews) | 29 patients | Convenience - patients of Sidney Kimmel Cancer Center at Thomas Jefferson University Hospital | Prostate | Mixed | 55 (Mean) 45-70 (Range) |
| **Wang** | 2020 | Quasiexperimental - control group received routine care and education, experimental group received 20 minutes education and guidance re: mHealth app (i.e. how to download, install, and use) | 100 patients | Convenience - patients of Far Eastern Memorial Hosptial, New Tapei City, Taiwan | Oral | Surgery | 57.01 (Mean) 8.87 (SD) |

| **First Author** | **Publication Date** | **Features** | **Availability** | **Stakeholder input** | **Patient preferences** |
| --- | --- | --- | --- | --- | --- |
| **Birkhoff** | 2018 | Appointment calendar Medication tracker Symptom tracker Journaling Daily mood diary Weight tracking Circle of support Vital sign tracker Healthy doses Tool library To-do list | Free and publicly available through developer website | App was previously designed and is available for a wide variety of patients - not oncology exclusive | Reported usability - overall 4.69, considerably higher among high school educated (6.38) versus grad school educated (3.87): Reported acceptability - there was no significant difference in app usage over time (i.e. no increase in uptake) |
| **Fishbein** | 2017 | Homepage (medical treatment plan and healthy recipes) Symptom reporting Symptom reporting trends Education library Notes and questions Wearable fitness tracking device | Not specified |  | Conducted but did not report usability/acceptability: Did implement features in app design based on stakeholder feedback through focus group and alpha/beta testing |
| **Greer** | 2020 | Personalized medication dosing schedule (with optional reminders) Adherence and symptom reporting Patient education (for symptom management and other cancer-related topics) Fitbit integration (for tracking physical activity) | Patients of Massachusetts General Hospital Cancer Center | Key stakeholders (patients, clinicians, healthcare system) | Did not report usability/acceptability or patient preferences |
| **Jacobs** | 2019 | Homepage Overview Local resources Treatments Day-to-day matters Health and wellbeing Social support Emotional support Favorites Settings | Publicly available - Google Play Store | cancer survivors and healthcare professionals | Tested usability average usefulness score was 4.2/5; patient engagement with app tasks was high, but several improvements were suggested - such as greater integration with local support services |
| **Kongshaug** | 2021 | Supporting adherence to medication (calendar view of medication plan, alerts and reminders for dose schedule) Management and reporting of side effects (with integral patient decision support system to call nurse at cancer clinic if needed, summary of all side effects registered in each treatment cycle) |  | Information and technology communication system developers and designers, 10 colleagues of the research team | App provided patients wth reassurance re: correct oral chemo treatment; app was used as a memory tool for med adherence and side-effects; patients were concerned about reporting less serious side-effects; health personnel expressed positive attitude to integrate tool in everyday work |
| **Tran** | 2020 | Notices of privacy and data usage for study purposes Symptom tracker Reminders to track symptoms (optional) | study only | not specified | Patients valued emtional and welbeing support over symptom reporting; patients requested incorporating patient online communities of support (such as Facebook or Reddit); patients were concerned with future data use and privacy; patients requested data summary features to help them track the information they were entering over time |
| **Wang** | 2020 | Latest news (latest communications to patients after surgery, links to YouTube videos on oral health education and head and neck rehab, link to patient groups through LINE app) Medical information (info on oral cancer, oral cancer treatment, pain information, hospice care, other supporting personnel or cancer treatment institutions) Self-recording (enables patients to record their own postoperative info and symptoms e.g. date, body temp, pain level, oral ulcer, vomiting, skin reactions, diarrhea) Revisit reminders | Not specified | not specified | acceptability of the app was measured through intention to use, perceived usefulness, and perceived ease of use (TAM model); baseline scores for experimental group were 2.54, 2.52, and 2.32; after intervention intervention group scores were 3.02, 2.95, and 3.01 - acceptability significantly increased in all three aspects of TAM; patients reported greater reliance on app information over leaflets distributed by health staff (boring and cumbersome) |
